# Interleukin-6 Secretion by Astrocytes Is Dynamically Regulated by PI3K-mTOR-Calcium Signaling

**DOI:** 10.1371/journal.pone.0092649

**Published:** 2014-03-25

**Authors:** Simone Codeluppi, Teresa Fernandez-Zafra, Katalin Sandor, Jacob Kjell, Qingsong Liu, Mathew Abrams, Lars Olson, Nathanael S. Gray, Camilla I. Svensson, Per Uhlén

**Affiliations:** 1 Laboratory of Molecular Neurobiology, Department of Medical Biochemistry and Biophysics, Karolinska Institutet, Stockholm, Sweden; 2 Department of Physiology and Pharmacology, Karolinska Institutet, Stockholm, Sweden; 3 Department of Neuroscience, Karolinska Institutet, Stockholm, Sweden; 4 Department of Cancer Biology, Dana-Farber Cancer Institute, Boston, Massachusetts, United States of America; 5 Department of Biological Chemistry and Molecular Pharmacology, Harvard Medical School, Boston, Massachusetts, United States of America; Hertie Institute for Clinical Brain Research, University of Tuebingen, Germany

## Abstract

After contusion spinal cord injury (SCI), astrocytes become reactive and form a glial scar. While this reduces spreading of the damage by containing the area of injury, it inhibits regeneration. One strategy to improve the recovery after SCI is therefore to reduce the inhibitory effect of the scar, once the acute phase of the injury has passed. The pleiotropic cytokine interleukin-6 (IL-6) is secreted immediately after injury and regulates scar formation; however, little is known about the role of IL-6 in the sub-acute phases of SCI. Interestingly, IL-6 also promotes axon regeneration, and therefore its induction in reactive astrocytes may improve regeneration after SCI. We found that IL-6 is expressed by astrocytes and neurons one week post-injury and then declines. Using primary cultures of rat astrocytes we delineated the molecular mechanisms that regulate IL-6 expression and secretion. IL-6 expression requires activation of p38 and depends on NF-κB transcriptional activity. Activation of these pathways in astrocytes occurs when the PI3K-mTOR-AKT pathway is inhibited. Furthermore, we found that an increase in cytosolic calcium concentration was necessary for IL-6 secretion. To induce IL-6 secretion in astrocytes, we used torin2 and rapamycin to block the PI3K-mTOR pathway and increase cytosolic calcium, respectively. Treating injured animals with torin2 and rapamycin for two weeks, starting two weeks after injury when the scar has been formed, lead to a modest effect on mechanical hypersensitivity, limited to the period of treatment. These data, taken together, suggest that treatment with torin2 and rapamycin induces IL-6 secretion by astrocytes and may contribute to the reduction of mechanical hypersensitivity after SCI.

## Introduction

The physiological outcome after spinal cord injury (SCI) is the result of a coordinated response of many cell types. Astrocytes play a key role in the scar formation that follows SCI [1]. During this process, astrocytes interact with microglia and immune cells to isolate and clear damaged tissue and to reestablish normal homeostasis of the spinal cord [2], [3]. In order to communicate with each other and regulate the surrounding environment these cells secrete cytokines [4]. Interestingly, the same signaling molecules can be secreted by different cell types at different time points after injury [5].

Interleukin-6 (IL-6) is a pleiotropic cytokine and its effects on SCI depend mostly on the temporal expression and the balance between survival-promoting and pro-inflammatory effects. Following SCI, microglia and macrophages secrete IL-6, which is thought to play a negative role in regeneration by recruiting immune cells to the site of injury and by promoting glial scar formation [6]. However, IL-6 expression also has positive roles in regeneration by promoting synaptic rearrangements, axon sprouting, and reducing tissue loss [7], [8].

In order to implement its function, IL-6 needs to be released into the extracellular space; hence, regulation of transcription-translation as well as of secretion are important for IL-6 mediated responses [9]. The Nuclear Factor-κB (NF-κB) is a strong inducer of IL-6 mRNA [10]. Various signaling cascades intersect with NF-κB to tightly regulate its activity [11] For example, the mitogen activated protein kinase (MAPK) p38, the phosphoinositide-3-kinase (PI3K) and the mechanistic target of rapamycin (mTOR) pathways. While activation of p38 promotes IL-6 expression, both PI3K and mTOR can exert inhibitory effects, depending on the cell type examined [12], [13].

After synthesis, IL-6 accumulates in secretory vesicles that upon stimulation fuse with the plasma membrane, releasing IL-6 into the extracellular space [9]. Increased intracellular calcium (Ca^2+^) is required for exocytosis. In cells, the endoplasmic reticulum (ER) is the main storage of intracellular Ca^2+^, which can be released into the cytoplasm through inositol-1,2,5-tris-phosphate receptors (InsP_3_R) or ryanodine receptors (RyR) [14]. Both receptor types are regulated by accessory proteins, such as the FK506-binding proteins (FKBP)-12 [15]. FKBP12 inhibits RyR mediated Ca^2+^ release while its effect on InsP_3_Rs is cell dependent and can either be to promote or inhibit Ca^2+^ release [16]–[18]. Interestingly, patients harboring mutations that increase the leakiness of RyRs show increased IL-6 secretion [19].

Although it has been shown that astrocytes secrete IL-6 [20], the signaling pathways involved are not well characterized. Hence, this study aims to understand which signaling pathways are important in the regulation of IL-6 in astrocytes in order to identify or develop drugs that can be used to up-regulate and/or down-regulate its secretion *in vivo*.

## Experimental Procedures

### Antibodies and other Reagents

#### Antibodies

The following commercially available antibodies (Cell Signaling Technologies) were used: pERK (#9101), pAKT-S473 (#9271), pAKT-T308 (#2965), AKT (#9272), Cleaved caspase-3 (#9661), GAPDH (#2118), pS6(S235/236) (#4858), p-p38(#8690), pJNK (#4668). IL-6 (Abcam, ab6672), Aldh1L1 (NeuroMab, N103/31), Vimentin (V6630) and GFAP (G-3893) (Sigma), ED1 (MCA341R) and OX42 (MCA275R) (Serotec), and NeuN (Chemicon MAB377). Secondary antibodies for immunohistochemistry and fluorescent western blotting (Life Technology), and HRP-conjugated secondaries (Sigma) were used.

#### Drugs

Rapamycin was commercially available (LC laboratories) as was LY294002 and FK506 (Tocris), the AKT inhibitor (Calbiochem) and Bapta-AM (Life Technology). Torin1 and torin2 were synthesized by Dr. Liu (Department of Cancer Biology, Dana-Farber Cancer Institute, Boston, MA).

#### Reagents

These included Complete mini (protease inhibitor) and phosphostop (phosphatase inhibitor) (Roche). All other reagents were from Sigma.

### Plasmids

pTK-RL (Stratagene), p1168huIL6P-Luc+ (wt IL-6 promoter, LMBP 4495) and p1168hIL6mNFκB-Luc+ (NFκB mutated promoter, LMBP 4496) (Belgian Coordinated Collections of Microorganisms (BCCM/LMBP)), and pRIL6C.94 (ATCC, ATCC-37681) were used.

### Astrocyte Cultures

Astrocyte cultures were prepared from spinal cords of adult male Sprague-Dawley rats (P65 to P70, weighing 200–250 grams Scanbur) using a method previously described [21]. For western blotting and real-time PCR, the cells were trypsinized and replated in 6-well plates (40,000 cells/well). The cultures were used for experiments when confluent (typically within 4–6 days). For mesoscale experiments, the cells were replated in 24-well plates (20,000 cells/well) and used when confluent (typically 2 days after plating). Before drug treatment the cells were incubated for 48 hours in growth-factor-free Dulbecco’s modified Eagle’s medium (GF Free DMEM), supplemented with 1% penicillin-streptomycin and 1% sodium pyruvate (Life Technologies).

### Spinal Cord Contusion Injury

Animal work was approved by the North Stockholm Animal Ethics Committee (permit # N429/09 and N479/11). Female rats (220 g, Scanbur, Sweden) were group-housed 4 animals per cage, and kept on a 12-hour light/dark cycle with food and water *ad libitum*. Spinal cord injury was done as previously described [22] with the Keck Center for Neurosciences impactor (NYU impactor) using a 10 g weight dropped from a height of 25 mm onto the dorsal surface of the exposed spinal cord. After recording BBB scores (see below), withdrawal thresholds evoked by touch stimulus, and body weights for the first week post-injury, animals were divided into 5 treatment groups: naïve (N = 4), sham (N = 6), vehicle (N = 6), torin2 (N = 6), and torin2+rapamycin (N = 8). Torin2 alone (4 mg/kg) or in combination with rapamycin (1.5 mg/kg) was administered orally by gavage once a day starting at day 15 after injury and ending at day 29. In sham operated rats only the laminectomy was performed. For all of the following experimental procedures and analyses, including behavioral testing and histological analyses, experimenters were blinded to the treatment groups.

### Locomotor Evaluation

The Basso, Beattie, Bresnahan (BBB) locomotor rating scale [23] was used to evaluate hindlimb locomotor function in an open field on a weekly basis for 6 weeks. All experimenters involved with BBB evaluation were blinded to treatment group identity.

### Assessment of Mechanical Hypersensitivity

Rats were habituated to the testing enclosure twice before the experiment started. Mechanical hypersensitivity was assessed three times prior to the surgery, once two weeks post-surgery to confirm the decrease in hind paw withdrawal threshold, and six times during the drug-treatment period on weeks 3–6 post-injury. Rats were placed in individual Plexiglas chambers on top of a wire mesh surface and allowed to acclimatize for 30 minutes prior to testing. Hind paw withdrawal thresholds were assessed by using calibrated von Frey filaments (Stoelting) with logarithmically incremental force of 0.4, 1, 2, 4, 6, 8, 10 and 15 g. A cut-off of 15 g was applied in order to avoid tissue damage, thus supra-threshold responses were not registered. Each filament was pressed perpendicularly against the plantar surface of the hind paw and held on for approximately 3 seconds. A positive response was recorded if the paw was withdrawn. The filaments were used according to the “up-down” method [24] and the 50% probability of withdrawal threshold described by Chaplan and co-workers [25] was calculated in grams for each hind paw and averaged. Mechanical hypersensitivity, the percent change from the average of the baseline values was also calculated.

### Tissue Processing

Naïve and injured rats at 6 hours, 1, 2, and 3 weeks after SCI (N = 4 for each condition) were anaesthetized with 50 mg/kg pentobarbital and perfused with 50 ml of calcium free Tyrode’s solution containing 0.1 ml heparin (5000 IE/ml) followed by 50 ml of 4% formaldehyde in PBS (PFA). For in situ hybridization and immunohistochemistry, spinal cords were dissected and post-fixed in 4% PFA at RT for 1 h and stored in 10% sucrose in PBS at 4°C. Spinal cords were then cut in 7 mm segments.

### In situ Hybridization

RNA probes for rat IL-6 mRNA were prepared as previously described [26]. IL-6 mRNA *in situ* hybridization was performed in spinal cord sections of naïve and injured animals at 6 hours, 1, and 2 weeks after SCI. Briefly, sections were permeabilized in PBST (PBS, 0.3% Tween-20), followed by overnight hybridization at 45°C with 10 ng/μl of riboprobe in hybridization solution (4 mM Tris-HCl pH 7.5, Poly-A 0.5 mg/ml, salmon sperm 0.5 mg/ml, 50% formamide, 20% dextran sulphate, 0.1 M DTT, 0.3 M NaCl, 1X Denhardt’s solution, 1 mM EDTA pH 8.0). Next, sections were washed twice for 15 min in 2X SSC at 45°C, 4×15 min with 0.1X SSC at 55°C, once for 10 min at RT in 5X MAB (2.5 M maleic acid; 3.75 M NaCl; pH 7.5), and three times for 10 min at RT in 1X MABT (0.5 M maleic acid; 0.75 M NaCl; 0.1% Tween-20; pH 7.5). Sections were then incubated with blocking solution (1X MAB, 20% donkey serum, 2% blocking agent; 0.1% Tween-20) for 1 h at RT followed by overnight incubation at RT with anti-digoxigenin antibody conjugated to alkaline phosphatase (Roche) (1∶2000) in blocking solution. After washing 4×20 min in 1X MABT and 3×10 min in B3 (0.1 M Tris-HCl, pH 9.5; 0.1 M NaCl; 50 mM MgCl2; 0.1% Tween-20), sections were developed for 4 h with NBT/BCIP (Roche) (1∶50) in B3 solution, and images were acquired (Olympus FV1000 CLSM Olympus).

### Immunohistochemistry

**Figure 1:**
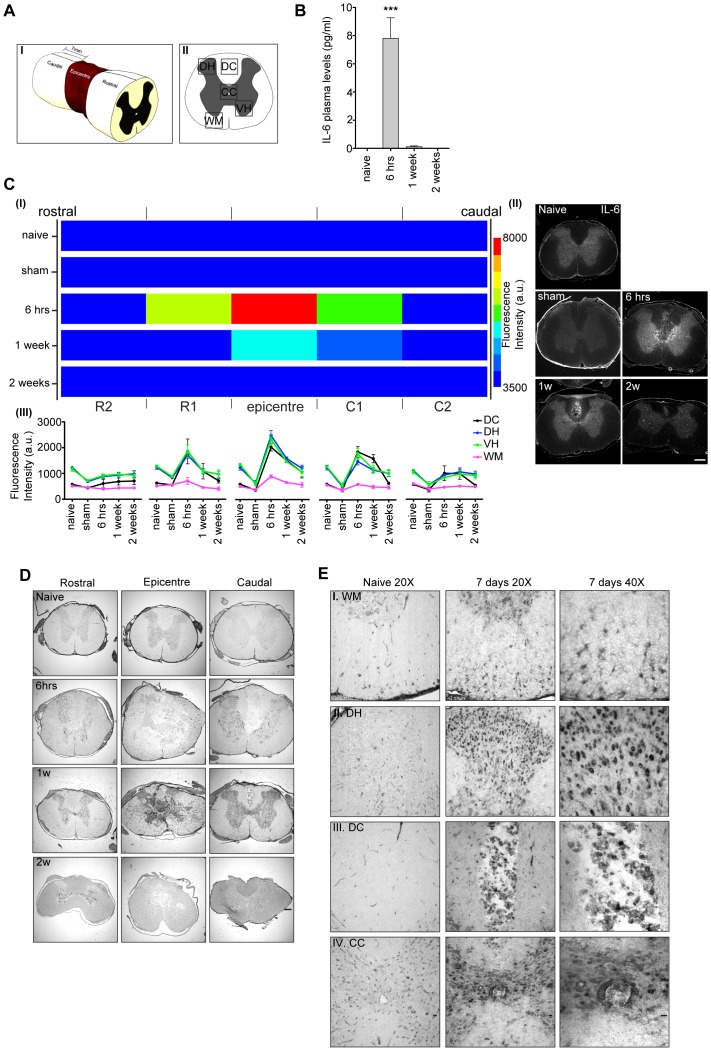
IL-6 is expressed in the spinal cord after contusion injury. After spinal cord contusion injury, IL-6 is upregulated in cells of the spinal cord. **A**, Cartoon depicting: (I) the 7 mm long segments (rostral, caudal, epicenter) in which the injured spinal cord is dissected for analysis. (II) Regions of the spinal cord sections imaged. Dorsal horn (DH), dorsal column (DC), central canal (CC), white matter (WM) and ventral horn (VH). **B**, IL-6 levels in plasma of animals used for *in situ* hybridization and immunohistochemistry. Data are presented as mean ± SEM. ***p<0.001 for the comparison between naive and injured animals by one-way ANOVA with Bonferroni’s *post hoc* test (N = 4 per group). **C**, (I) Representation of the IL-6 protein distribution quantified by immunohistochemistry in spinal cord segments at different time points after injury. Blue indicates the lowest and red the highest intensity of IL-6 immunofluorescence (arbitrary units, a.u.). R, rostral and C, caudal. (II) Representative images of IL-6 labeled sections from the C1 region of naïve, sham (1 week after injury) and injured animals. Scale bar = 20 μm. (III) Quantification of IL-6 immunoreactivity in different regions of the spinal cord at different time points after injury. N = 3 animals for each condition. For each spinal cord segment three sections have been imaged and fluorescence signal in the regions defined in A(II) was quantified and averaged in each section. **D**, *In situ* hybridization with an antisense IL-6 mRNA probe on sections from rostral, epicenter and caudal segments of spinal cord from naive and injured animals 6 hours, 1, and 2 weeks post-injury. Scale bar = 200 μm. **E**, High magnification images from sections of the caudal segment shown in D from naive (20X) and 1 week injured spinal cord animals (20X and 40X). Scale bar = 20 μm for 20X and 40X. (N = 3 in each group).

Spinal cord sections were permeabilized twice for 5 min with TBST (TBS, 0.2% Tween-20), blocked for 1 h at RT in blocking solution (TBST with 5% goat serum), incubated overnight at 4°C with primary antibodies in blocking solution, and finally incubated for 1 h at RT with Alexa-conjugated secondary antibodies (Life Technologies) in blocking solution. DAPI staining was applied for 30 min in blocking solution. Images were acquired using confocal microscopy (Olympus FV1000 or Zeiss LSM700). Imaris 7.3.0 (Bitplane) was used to quantify the intensity of IL-6, pS6 and pS6/IL-6 colocalization in GFAP positive cells. In order to identify GFAP positive cells we first rendered the image stack in 3 D. The GFAP channel was then selected and the pixels above a minimal threshold were used to build a 3 D surface. This 3 D surface isolated the GFAP positive cells. IL-6 and pS6 intensity and pS6/IL-6 colocalization were quantified inside this GFAP positive surface. The settings used in the image processing were the same for all images analyzed. The figures were prepared using appropriate image handling software (Adobe Illustrator CS5, Adobe Systems). IL-6 immunofluorescence in the different regions and segments of the spinal cord was quantified by measuring the integrated intensity of ROIs selected (Fiji) [27]. The heat map in Figure 1C was generated using a custom script in python/matplotlib.

### Real-time PCR

All reagents used were commercially available (Life Technologies). Probe and primer for IL-6 mRNA were “assay-on-demand” gene expression products (TaqMan Probe and primer ID Rn00561420_m1). After stimulation with drugs (8 hrs), astrocytes were washed with PBS and lysate using Trizol. Total RNA was purified using phenol-chloroform and reversed transcribed using random hexanucleotide primers. PCR amplification reactions were carried out in 25 μl containing 50 ng of cDNA (MicroAmp Optical Plates with MicroAmp Optical Caps and the TaqMan Universal Master Mix). Incubations at 50°C for 2 min and at 95°C for 10 min were carried out to activate the polymerase (AmpliTaq polymerase), followed by 40 cycles at 95°C for 15 s and 60°C for 1 min. GAPDH was used as a loading control for each sample and the standard curve method was used for quantification [28].

### Immunoblotting

Astrocyte cultures were lysed in RIPA buffer (Sigma) containing protease inhibitors and phosphatase inhibitor cocktails. Samples were analyzed by SDS-PAGE followed by immunoblotting. Membranes were incubated with primary antibodies followed by horseradish peroxidase-conjugated secondary antibodies and ECL prime chemiluminescence detection reagent (GE). Chemiluminescent signal was detected using a Biorad ChemiDocXRS+ (Biorad). For detection using near-infrared, membranes were incubated with secondary antibodies (Alexa-680 and Alexa-790) and scanned (Odyssey scanner, LICOR). Membranes were stripped (ReBlot Western Blot Recycling Kit, Millipore) before re-probing with a different antibody.

### Transfection and Luciferase Gene Reporter Assay

In reporter gene assays, 10^6^ cells were transiently transfected (Amaxa nucleofection, VPI-1006, electroporation program T-020) according to the manufacturer’s instructions. Astrocytes were transfected with 400****ng of the pRL-TK-Luc plasmid together with 3600 ng of p1168huIL6P-Luc+ or p1168hIL6mNFkB-Luc+ plasmid and plated in complete media for 24 hrs. After 24 hours, the media was replaced with GF-free media for an additional 24 hours. Drugs were then applied and after 24 hours the cells were lysed in reporter lysis buffer (Promega). Reporter activity was determined (Promega dual luciferase assay system) according to manufacturer’s instructions. Firefly and Renilla activities were detected (Victor^2^ reader, Wallac). Firefly luciferase values were normalized for transfection efficiency by means of the Renilla luciferase activity that is constitutively expressed by pRL-TK.

### Cytokine Detection

High sensitivity detection of rat IL-6 was carried out using a commercially available kit (KC153AKC-1 rat IL-6 ultra-sensitive kit, Mesoscale discovery, MSD). For cytokine detection in cell culture supernatant, 20,000 cells/well were plated in 24-well plates and incubated 2 days until confluent. Culture media was then replaced with GF-free media. After 48 hours, the GF-free media was replaced with GF-free media containing drugs. After 24 hours, the supernatant was collected and centrifuged at 4°C 10,000 rpm for 5 minutes to remove cell debris. The supernatant was collected without disturbing the pellet, aliquoted and frozen on dry ice. For analysis of cytokines in plasma, blood was collected by cardiac puncture before (naive), 6 hours, 7 days, and 14 days after injury. The blood was collected in micro container SSB tubes (BD) and the plasma was separated by centrifugation at 6000 rcf for 90 seconds. After isolation, the plasma was frozen on dry ice.

For cytokine detections in supernatant and plasma samples, we followed the manufacturer’s instructions with minor modification. The 96-well plates were incubated with diluent solution with 2% BSA (25 μl) for 30 minutes at RT on an orbital shaker. Samples and standards were added (25 μl) and the plate was incubated over night at 4°C on an orbital shaker. The wells were then washed three times using 200 μl PBS +0.05%Tween-20. The detection antibody was added and the plate was incubated for 2 hrs at room temperature on an orbital shaker. At the end of the incubation the plate was washed three times using 200 μl PBS +0.05%Tween-20. Read Buffer (150 μl, MSD) was then added to each well and IL-6 was measured (MSD Sector Imager 2400 plate reader). Raw data were analyzed using MSD software (Discovery Workbench 3.0). A 4-parameter logistic fit curve was generated using the standards and the concentration of each sample calculated.

### Calcium Measurements

After 48 h starvation and 24 h treatment with torin2 and/or rapamycin, spinal cord astrocytes were loaded with 5 μM Fura2-AM and 0.1% Pluronic Acid (F-127 Life Technologies) for 30 min at 37°C in starving media, rinsed twice, and imaged in Ca^2+^-free KREBS-Ringer’s solution (119 mM NaCl, 2.5 mM KCl, 1 mM NaH2PO4, 2 mM EGTA, 1.3 mM MgCl2, 20 mM HEPES, 11 mM D-glucose) at 37°C. After baseline readings, 3 mM of 4-chloro-methylphenol (4-cmc) was bath-applied to the cells. mTOR inhibitors were present during loading and throughout the duration of the experiment. Images were acquired at 340 nm and 380 nm at a frequency of 0.5 Hz using an upright widefield microscope (Zeiss) equipped with a 20x water immersion objective. Image analysis was done using custom Python scripts.

### In situ Detection of Fragmented DNA (TUNEL Assay)

Astrocytes were plated in poly-lysine-coated plates with transparent bottom for high content screening (BD). After 24 hours, the cells were incubated in GF-free media and 48 hours later the cells were incubated with GF-free media containing the drugs. After 24 hours incubation, fragmented DNA was detected using a commercially available kit (Click-iT TUNEL Alexa Fluor Imaging Assay, Life Technology) as indicated by the manufacturer. The plate was imaged using a cell observer (Zeiss) and the percentage of TUNEL-positive cells was quantified with a custom pipeline generated in CellProfiler [29].

## Results

### Reactive Astrocytes Express IL-6 after Contusion Spinal Cord Injury

In order to examine IL-6 expression in the spinal cord after injury, we used a contusion injury model. The corticospinal tract and other dorsal parts of the spinal cord were damaged by dropping a weight on the exposed dura using the NYU impactor [23], [30]. We collected spinal cords and plasma from animals at 6 hours, 1, 2, and 3 weeks after injury, control cords were obtained from sham operated and naïve rats. It has been shown that strong astrocyte activation is detectable 1 week after contusion injury; however, the astrocyte scar is fully formed 2–3 weeks post-injury [31]. Interestingly, IL-6 levels in plasma were already high 6 hours after injury, but returned to basal by week one (Figure 1B). We next examined spinal cord sections from the same animals by *in situ* hybridization and immunohistochemistry. In order to determine if IL-6 expression in the spinal cord correlated with the plasma levels, we divided the spinal cords in 7 mm-long segments and quantified IL-6 expression by immunohistochemistry (Figure 1A,C). We found that the IL-6 level was highest at 6 hours after injury and returned to baseline by week two (Figure 1C (I,II)). Noteworthy, sham surgery (1 week) did not cause an increase of IL-6 levels in the spinal cord compared to levels in naïve animals (Figure 1C). Moreover, IL-6 protein was not homogenously expressed along the spinal cord length; IL-6 was higher in the epicenter segment while levels were lower rostrally and caudally (Figure 1C (I)). Interestingly, when the different regions in the spinal cord sections were examined, IL-6 expression increased in the gray matter (dorsal and ventral horn) and in the dorsal column, but no changes were detectable in other regions (Figure 1C (III)).

In order to determine if cells of the injured spinal cord produce IL-6, we used *in situ* hybridization to detect IL-6 mRNA in tissue sections (Figure 1D–E). An increase of IL-6 mRNA was detectable in the injury epicenter, both dorsal and caudal to the injury site at 6 hours, 1, and 2 weeks after injury. These areas showed a marked upregulation of IL-6 mRNA in cells of gray and white matter of the injured spinal cord as compared to naïve animals (Figure 1D–E). An IL-6 sense *in situ* probe was used as a control and specificity was indicated by lack of signal in naïve tissue and in tissue harvested 1 and 2 weeks after injury (Figure S1). High magnification images of spinal cord sections showed that IL-6 mRNA was expressed in different cell types (Figure 1E).

**Figure 2:**
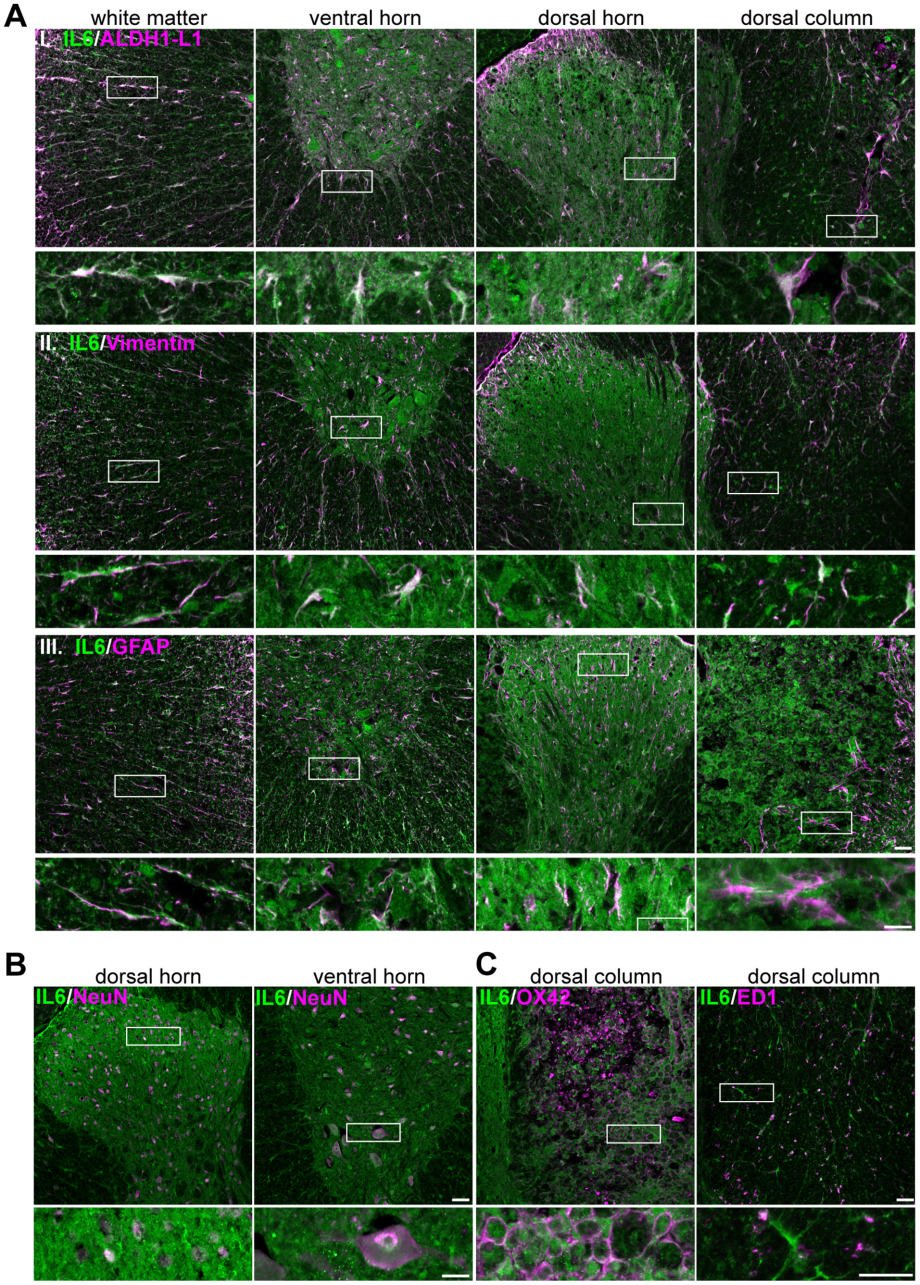
Astrocytes express IL-6 1 week after contusion injury. Reactive astrocytes express IL-6 after contusion injury as well as neurons and microglia/macrophages. **A**, Immunolabeling of IL-6 (green) and astrocyte markers (magenta) ALDH1L1 (I), vimentin (II) and GFAP (III) in different regions of the caudal segment from spinal cords 1 week after injury. Scale bar = 20 μm. **B**, Immunolabeling of IL-6 (green) and the neuronal marker NeuN (magenta) in dorsal and ventral horns of caudal segments from spinal cords 1 week after injury. Scale bar = 20 μm. **C**, Immunolabeling for IL-6 (green) and the microglia and macrophage markers OX-42 and ED-1 (magenta) in the dorsal column of the caudal segment from spinal cords 1 week after injury. Scale bar = 20 μm. N = 3 for all conditions.

To determine if astrocytes express IL-6, we assessed the colocalization of IL-6 immunoreactivity with the pan-astrocytic marker aldehyde dehydrogenase 1 family member L1 (ALDH1L1) and markers of reactive astrocytes, such as vimentin and glial fibrillary acidic protein (GFAP). We found that IL-6 protein expression colocalized with all three astrocyte markers in all regions of the spinal cord examined (Figure 2A). However, our *in situ* data showed that at 1 week post-injury, IL-6 mRNA was present in multiple cell types (Figure 1E). Hence, to investigate if IL-6 was also produced by neurons and/or microglia and macrophages, we determined the degree of colocalization between IL-6 and the neuronal marker NeuN and between IL-6 and the microglia/macrophage markers OX42, and ED1. We found that IL-6 was not only expressed by reactive astrocytes but also by neurons of the dorsal and ventral horn (Figure 2B) as well as by macrophages and microglia in the dorsal column (Figure 2C). However, at 1 week after injury, macrophages or microglia did not express IL-6 in the white matter or in any of the other regions of the spinal cord analyzed (Figure S2).

**Figure 3:**
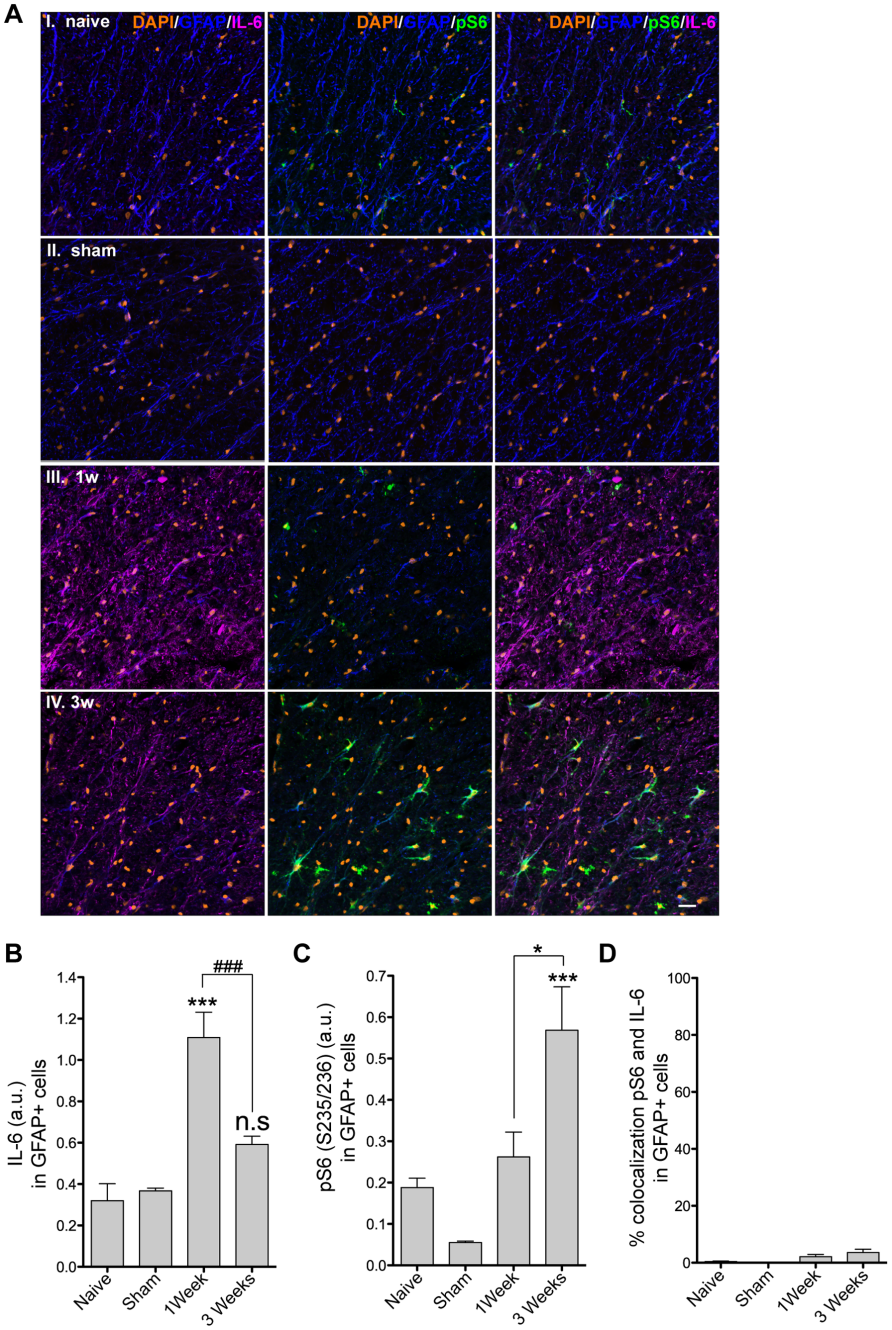
mTORC1 activity and IL-6 expression are negatively correlated in SCI. IL-6 expression in reactive astrocytes of the white matter returns to baseline three weeks after injury, at a time when mTORC1 activation peaks. **A**, Immunolabeling for pS6 (235/236) (green), IL-6 (magenta), GFAP (blue), and DAPI (orange) in white matter of the caudal segment of spinal cords from naïve (I), sham (II) and injured animals 1 (III) and 3 (IV) weeks after injury. For all images, colocalization between IL-6 and pS6 is depicted in white. Scale bar = 20 μM. **B**, Quantification of IL-6 immunofluorescence in GFAP+ astrocytes in white matter of the caudal segment of the spinal cord from naïve and sham animals and animals 1 and 3 weeks after injury. In each section IL-6 immunofluorescence is normalized to the total GFAP fluorescence signal. Data presented as mean ± SEM. ***p<0.001 and n.s. (not significant) for the comparison between sham and injured animals and ### p<0.01 for the comparison between 1 week and 3 weeks after injury by one-way ANOVA and Bonferroni’s *post hoc* test (N = 3 in each group). **C**, Quantification of pS6 immunofluorescence in GFAP+ astrocytes from the white matter of sections from the caudal segment of the spinal cord from naïve and sham animals and animals 1 and 3 weeks after injury. In each section, IL-6 immunofluorescence is normalized for the total GFAP fluorescence signal. Data are presented as mean ± SEM (N = 3 in each group). ***p<0.001 for the comparison between sham and injured animals and * p<0.05 for the comparison between 1 week and 3 weeks injured animals by one-way ANOVA with Bonferroni’s *post hoc* test. **D**, Quantification of IL-6 and pS6 colocalization in GFAP+ astrocytes from the white matter of sections from the caudal segment of the spinal cord from naïve, and sham and animals, and animals 1 and 3 weeks after injury.

Because astrocyte properties change during the different phases of SCI [32], we examined if IL-6 immunoreactivity in astrocytes 1 and 3 weeks after injury (Figure 3A). We found that IL-6 expression in GFAP+ cells was significantly increased at 1 week (1.1±0.1 a.u.) and returned to sham levels (0.38±0.01 a.u.) at 3 weeks post-injury (0.59±0.04 a.u.), indicating that IL-6 was expressed at the beginning of the astrocyte response but returned to baseline when the scar was fully formed (Figure 3B).

### mTORC1 Activity and IL-6 Expression are Negatively Correlated in SCI

Recent reports showed that PI3K and mTOR inhibition regulate IL-6 expression in immune cells [13], [33]. Since mTORC1 is the main regulator of protein translation, we decided to examine if mTORC1 can regulate IL-6 expression in astrocytes following injury. As the mTORC1 activity readout, we measured the phosphorylation level of the downstream target ribosomal protein S6 by immunofluorescence in GFAP+ cells in tissue sections from spinal cord collected at 1 and 3 weeks after injury (Figure 3C). Interestingly, pS6 immunoreactivity negatively correlated with IL-6 expression in reactive astrocytes of the white matter. pS6 was low in astrocytes 1 week post-injury (0.26±0.06 a.u.), when IL-6 level was the highest (1.1±0.1 a.u.), and high at 3 weeks (0.57±0.1 a.u.), when IL-6 expression in astrocytes had returned to baseline (0.59±0.04 a.u.) (Figure 3B,C), suggesting that, *in vivo,* mTORC1 activity inhibits IL-6 expression. Moreover, no colocalization was detected between IL-6 and pS6 in GFAP+ astrocytes at any of the time points analyzed (Figure 3D).

### The PI3K-mTOR Pathway Regulates IL-6 Expression in Cultured Spinal Cord Astrocytes

**Figure 4:**
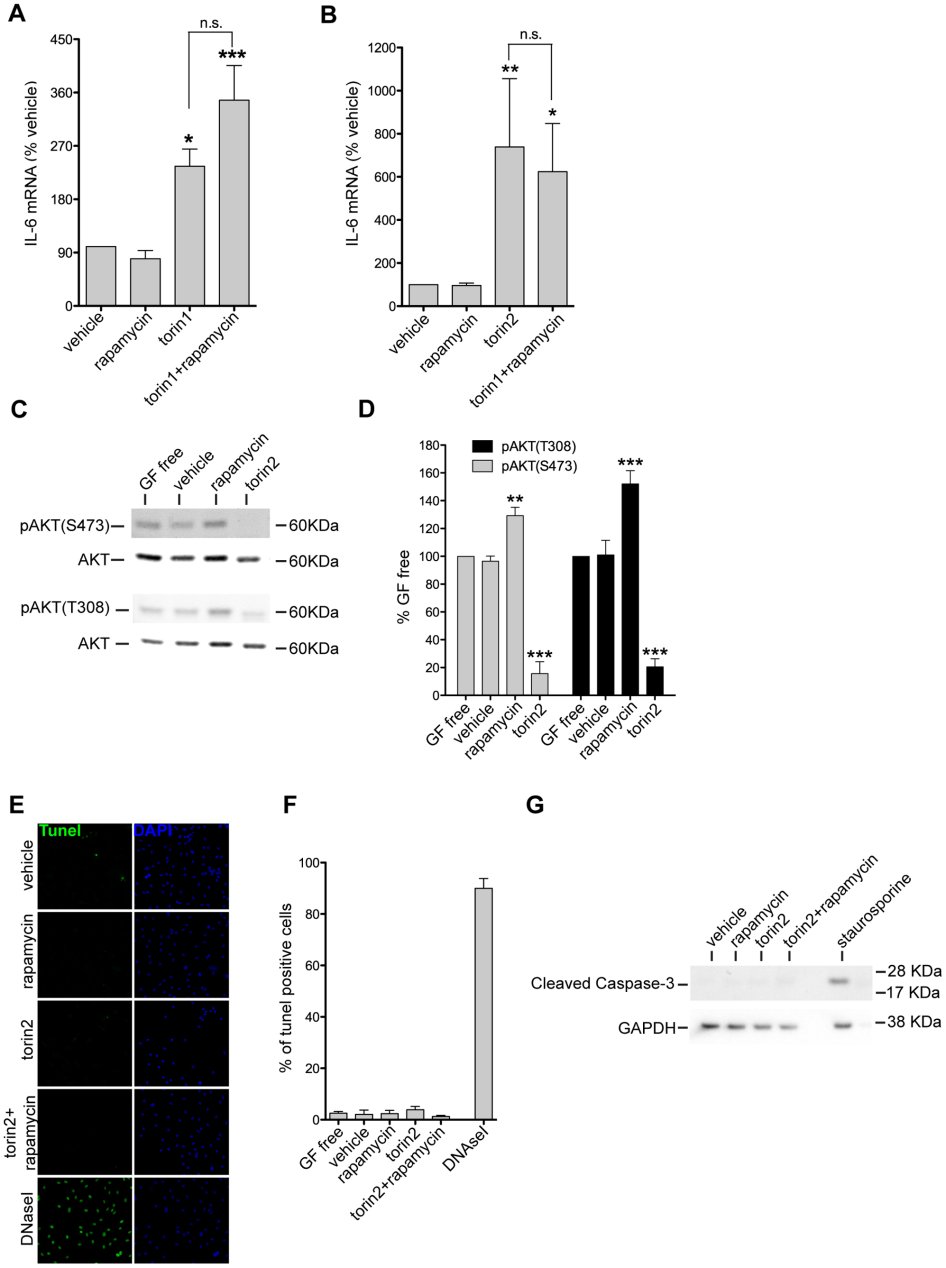
The PI3K-mTOR pathway regulates IL-6 expression in cultured spinal cord astrocytes. PI3K-mTOR inhibition with the ATP-competitive drugs, torin1 and torin2, inhibits AKT and induces IL-6 expression without increasing cell death in primary cultures of adult spinal cord astrocytes. **A,** Torin1 and rapamycin effects on IL-6 mRNA expression quantified by qPCR and expressed as percentage of vehicle treated cells. Data presented as mean ± SEM. *p<0.05 and ***p<0.001 for the comparison between vehicle and torin1 or torin1+rapamycin treated cells and n.s. (not significant) for the comparison between torin1 and torin1+rapamycin treated cells by one-way ANOVA with Bonferroni’s *post hoc* test (N = 4–5 per treatement). **B,** Torin2 and rapamycin effects on IL-6 mRNA expression quantified by qPCR and expressed as percentage of vehicle treated cells. Data presented as mean ± SEM. *p<0.05 and **p<0.01 for the comparison between vehicle and torin2 or torin2+rapamycin treated cells and n.s. (not significant) for the comparison between torin2 and torin2+rapamycin treated cells by one-way ANOVA with Bonferroni’s *post hoc* test (N = 4–5 per treatement). **C**, Lysates from untreated cells (GF free) and cell treated with vehicle, rapamycin, and torin2 were probed by immunoblotting with the indicated antibodies. **D**, Optical densities of phospho-AKT serine 473 and threonine 308 bands normalized to levels of AKT in the lysates. Average levels of phospho-AKT serine 473 and phospho-AKT threonine 308 shown as percentage of GF free condition. Data are presented as mean ± SEM. ***p<0.001 and **p<0.01 for the comparison between vehicle and drug treated cells by one-way ANOVA with Bonferroni’s *post hoc* test (N = 4–5 per treatement). **E**, Fragmented DNA labelled (tunel, green) in astrocytes treated with the indicated drugs or DNAseI as positive control. Astrocyte nuclei were visualized by DAPI (blue) staining. **F**, Numbers of tunel positive cells. **G**, Lysates from vehicle and cells treated with rapamycin, torin2, torin2+rapamycin, and staurosporine probed by immunoblotting with cleaved caspase-3 antibody and GAPDH.

To increase understanding of the mechanisms that regulate IL-6 production in astrocytes, we used cultured astrocytes from adult rat spinal cord. More than 99% of these cells express astrocytic markers [21]. To avoid IL-6 induction, all experiments were performed with cultures incubated in growth factor free media (GF free). Noteworthy, the amino acids present in the media were sufficient to activate PI3K and both mTORC1 and mTORC2 (Figure 4C–D, GF free condition) [34].

We stimulated primary astrocytes with rapamycin for 8 hours and measured IL-6 mRNA by qPCR in order to determine if mTORC1 inhibition can also promote IL-6 expression. We found that rapamycin had no effect on IL-6 mRNA levels (Figure 4A–B). Even though rapamycin inhibits mTORC1, in astrocytes it also induces activation of mTORC2 as well as the PI3K downstream target pyruvate dehydrogenase kinase, isozyme 1 (PDK1), as shown by an increased phosphorylation of AKT at serine 473 (mTORC2 dependent) and threonine 308 (PDK1 dependent) (Figure 4C–D). Therefore, in order to inhibit both mTORC1 and mTORC2, we treated astrocytes with torin1 and torin2, two analogues that block the ATP binding site of mTOR [35], [36]. Interestingly, both torin1 and torin2, at the concentration used (1 μM), inhibited mTORC1 and mTORC2, as well as AKT phosphorylation at threonine 308, suggesting inhibition of the PI3K-PDK1 pathway (Figure 4C–D and data now shown). Torin1 and torin2 treatment increased IL-6 mRNA, suggesting that the PI3K-mTOR pathway is a negative regulator of IL-6 expression in astrocytes (Figure 4A–B). Importantly, torin2 treatment did not show any cell toxicity, as no signs of cell death were observed by TUNEL assay or by detection of cleaved-caspase 3 by western blotting (Figure 4E–G).

### NF-κB and p38 Mediate the Effect of PI3K-mTOR on IL-6 Expression

**Figure 5:**
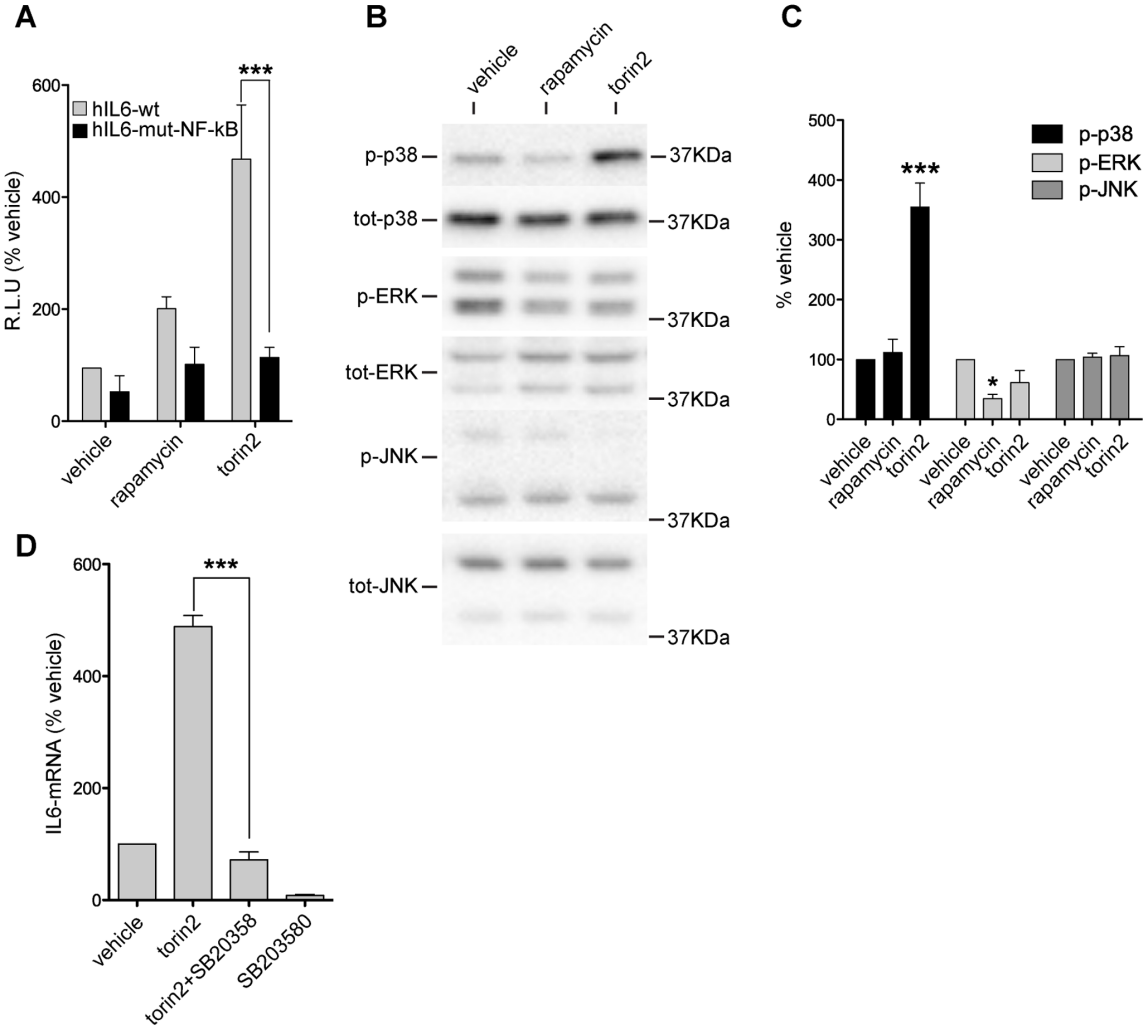
NF-κB and MAPKs mediate the effect of PI3K-mTOR on IL-6 expression. Astrocytes treated with torin2 have elevated MAPK signaling and express IL-6 in a NF-κB dependent manner. **A**, Bar graph showing luciferase activity (relative luciferase units R.L.U) expressed as percentage of luciferase activity in vehicle treated cells. The grey bars represent astrocytes transfected with a plasmid in which wild type hIL-6 promoter (hIL6-wt) drives luciferase expression. The black bars represent cells transfected with a plasmid in which a hIL-6 promoter with a mutated NF-κB binding site drives luciferase expression (hIL6-mut-NF-κB). Data are presented as mean ± SEM. ***p<0.001 for the comparison between wt and mut-NF-κB transfected astrocytes by two-way ANOVA with Bonferroni’s *post hoc* test (N = 3 per treatement). **B**, Lysates from vehicle, rapamycin, and torin2 treated cells probed by immunoblotting with antibodies for phosphorylated and not phosphorylated forms of p38, ERK, and JNK. **C**, Optical densities of the phospho-protein bands were normalized to the levels of the corresponding not phosphorylated proteins in the lysates and expressed as percentage of vehicle treated cells. Average levels of phospho-ERK, phospho-JNK, and phospho-p38 shown as mean ± SEM. ***p<0.001 and *p<0.05 for the comparison between vehicle and drug treated cells by one-way ANOVA with Bonferroni’s *post hoc* test (N = 3 per treatement). **D**, IL-6 mRNA expression quantified by real-time quantitative PCR and expressed as a percentage of expression in vehicle treated cells. Data are presented as mean ± SEM. ***p<0.001, for the comparison between torin2 and torin2+SB20358 treated cells by one-way ANOVA with Bonferroni’s *post hoc* test (N = 4–5 per treatement).

The transcription factor NF-κB is a strong inducer of IL-6 [10]; therefore, to determine if PI3K-mTOR inhibition induces IL-6 mRNA through NF-κB regulation, we transfected astrocytes with a plasmid containing the firefly luciferase gene under the control of either the wild type hIL-6 promoter (wt) or a hIL-6 promoter in which the binding site for NF-κB is mutated in order to prevent the binding of the transcription factor. It has been previously reported that both promoters are able to drive luciferase expression in the same way when stimulated by an NF-κB-independent stimulus [3], [37]. The plasmids were co-transfected with the co-reporter vector pRL-TK. Astrocytes transfected with the wt hIL-6 promoter showed a strong induction of luciferase signal after torin2 treatment (472.9±97.1%), confirming that inhibition of PI3K-mTOR regulates IL-6 mRNA transcription (Figure 5A gray bars). The mutation of the NF-κB binding site reduced the luciferase signal to baseline (119.2±17.8%), indicating that NF-κB regulates IL-6 transcription (Figure 5A). These data indicate that the PI3K-mTOR pathway can regulate IL-6 transcription through modulation of NF-κB activity in astrocytes.

Previous work has shown that NF-κB activation requires phosphorylation and degradation of the inhibitory subunit IκB by the IκB kinase (IKK). MAPKs can regulate IL-6 mRNA expression via phosphorylation and activation of IKK. Once active, IKK acts by phosphorylating and degrading NF-κB inhibitory subunit IκB, resulting in NF-κB activation [11]. In order to examine if MAPKs activation is increased after PI3K-mTOR inhibition, we treated primary cultures of astrocytes with torin2 and blotted for the activated form of p38, ERK1/2, and JNK (Figure 5B). Astrocytes maintained in GF free media have a basal level of p38, ERK1/2, and JNK activation, and torin2 treatment increased p38 phosphorylation (355±40%) (Figure 5C), suggesting that NF-κB activation may result from increased p38 activation. In order to assess if p38 mediates the effect of PI3K-mTOR on IL-6 expression, we treated primary cultures with the p38 inhibitor SB203580 (Figure 5D), and found that SB203580 treatment blocked the IL-6 mRNA induction produced by torin2 treatment, suggesting that p38 can regulate IL-6 mRNA expression in astrocytes (Figure 5D).

### PI3K-mTOR Inhibition and Increased Cytosolic Calcium are Necessary for IL-6 Secretion

**Figure 6:**
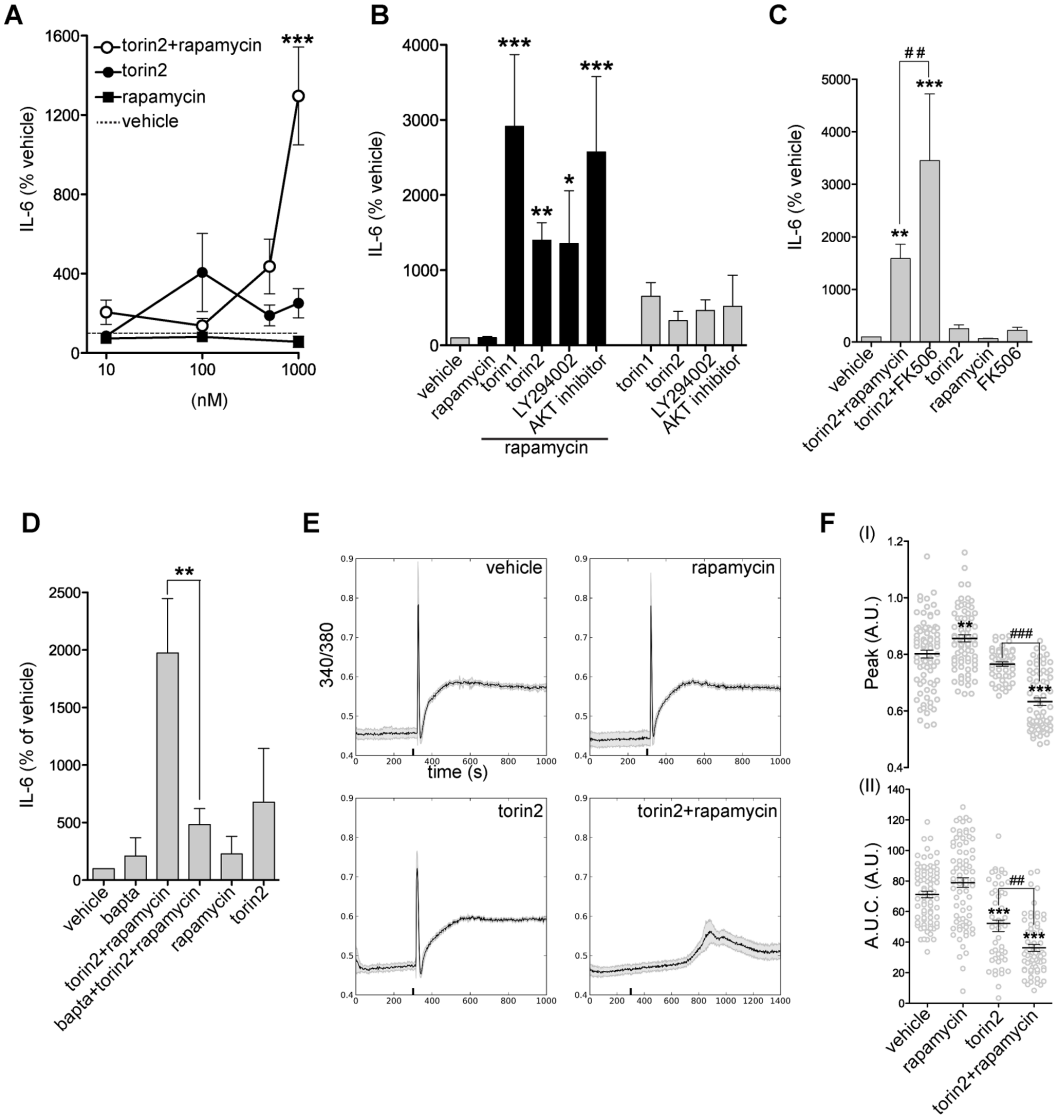
PI3K-mTOR inhibition and increased cytosolic calcium are necessary for IL-6 secretion. Combined treatment with torin2 and rapamycin induces IL-6 secretion by astrocytes as a result of PI3K-mTOR-AKT inhibition and increased intracellular calcium. **A**, Dose-response curve for rapamycin, torin2, and torin2+rapamycin treatments. IL-6 protein secretion in the supernatant was measured from cultures of astrocytes incubated with increasing concentrations (10 nM-1 μM) of torin2 or rapamycin alone or torin2 in combination with rapamycin (100 nM) (N = 3–7). ***p<0.001 for the comparison between torin2 (1 μM) and torin2 (1 μM)+rapamycin (100 nM) by one-way ANOVA with Bonferroni’s *post hoc* test. **B**, IL-6 protein levels in the supernatant of astrocyte cultures treated for 24 hours with vehicle, rapamycin, torin1, torin2, LY294002, and AKT inhibitor alone or in combination with rapamycin. Mean and SEM (N = 4–12 per treatement). *p<0.05, **p<0.01 and ***p<0.001 for the comparison between drug treated cells by one-way ANOVA with Bonferroni’s *post hoc* tests. **C**, IL-6 levels in the supernatant for cells treated with torin2, torin2+ rapamycin, torin2+ FK506, rapamycin, and FK506 (N = 3). **p<0.01 and ***p<0.001 for comparison between drug treated cells and vehicle and ##p<0.01 for the comparison torin2+rapamycin and torin2+FK506 by one-way ANOVA with Bonferroni’s *post hoc* tests. **D**, IL-6 protein levels in the supernatant of astrocyte cultures treated for 24 hours with vehicle, rapamycin, torin2, and rapamycin+torin2 with or without bapta-1am pre-treatment, expressed as percentage of levels in vehicle treated cells (N = 4 per treatement). **p<0.01 comparison between torin2+rapamycin and torin2+rapamycin+bapta treated cells by one-way ANOVA with Bonferroni’s *post hoc* tests. **E**, Representative Fura-2 calcium traces of 4-cmc treated cultures. Before stimulation cells were incubated 24 hours with vehicle, rapamycin, torin2, or torin2+rapamycin. The black bar at 300 (s) indicates the time of 4-cmc stimulation. Grey traces show the SEM of the response of all the cells analyzed in the experiment and the black trace corresponds to the average response. The torin2+rapamycin graph shows a longer time scale to highlight the delay in the response (n = 49–78 cells per condition in a total of N = 3 experiments). **F**, Quantification of response (I) and area under the curve (A.U.C) (II) after stimulation with 4-cmc (n = 49–78 cells per condition in N = 3 experiments). **p<0.01 and ***p<0.001 for the comparison between torin2 and torin2+rapamycin with vehicle treated astrocytes. ###p<0.001 and ##p<0.01 for the comparison between torin2 and torin2+rapamycin treated cells by one-way ANOVA with Bonferroni’s *post hoc* tests.

We hypothesized that in order to induce IL-6 secretion in primary cultures of astrocytes, more than one of the molecular steps involved in regulation of cytokine production would have to be manipulated. Our data indicate that IL-6 expression in astrocytes can be induced by PI3K-mTOR inhibition by torin2 (Figure 4B). It has been previously reported that increased cytosolic Ca^2+^ through leaky RyRs in the ER promotes IL-6 secretion [19]. Interestingly, even though rapamycin is commonly used as an mTORC1 inhibitor, it functions by binding FKBP12, a regulator of RyRs gating. Binding of rapamycin to FKBP12 results in increased open probability of RyRs and increased cytosolic Ca^2+^ concentration [15]. Hence, we induced IL-6 expression and affected RyRs by using a combined treatment of torin2 and rapamycin. We found that when astrocytes were treated with torin2/1+rapamycin, IL-6 was detected in the supernatant, but only when torin2 was used at 1 μM, the concentration at which also PI3K and PDK1-AKT pathways were inhibited (Figure 6A,C, 4B–C). Moreover, neither torin2 or rapamycin treatment alone was able to induce IL-6 protein secretion in the supernatant (Figure 6A), suggesting that in astrocytes an increase in gene expression and protein secretion are both necessary for IL-6 production. Noteworthy, IL-6 mRNA levels in torin1/2+rapamycin treated astrocytes were not statistically different from the increase caused by torin1/2 treatment alone, suggesting that the effect of rapamycin on IL-6 secretion is not caused by an enhanced IL-6 expression in the combined treatment (Fig. 4A–B).

To confirm that PI3K-mTOR inhibition is necessary for IL-6 secretion, we treated astrocytes with rapamycin together with the PI3K inhibitor LY294002 at a concentration of 30 μM, which is known to also inhibit mTOR [38]. Interestingly, LY294002+rapamycin induced IL-6 secretion (1355±703%) at a level comparable to torin2+rapamycin treatment (1399±234%) (Figure 6B). Since torin2 treatment caused AKT inhibition in astrocytes (Figure 4C–D), we examined if AKT could be mediating the effect of PI3K-mTOR in IL-6 secretion. While the AKT inhibitor alone had no effect on IL-6 secretion, the combination with rapamycin strongly induced IL-6 in the supernatant (2576±1001%), indicating that AKT mediates the PI3K-mTOR effect on IL-6 secretion (Figure 6B). To confirm that the effect of rapamycin on IL-6 secretion was independent from its inhibitory function on mTORC1, but depended on its regulation of RyRs permeability and cytosolic Ca^2+^ concentration, we treated cells with FK506 (Figure 6C). FK506 is a potent immunosuppressant that forms a drug-immunophilin complex with FKBP12, displacing it from the RyRs [15]. However, the FK506-FKBP12 complex is not able to interact and inhibit mTOR, and thus is often used as a control to monitor mTOR-independent rapamycin effects [39]. IL-6 secretion in astrocytes was not affected by treatment with FK506 (Figure 6C); however, FK506 in combination with torin2 caused a strong secretion of IL-6 (Figure 6C), confirming that IL-6 secretion depends on RyRs. In order to determine whether IL-6 secretion caused by torin2+rapamycin treatment was Ca^2+^ dependent, we pre-treated cell cultures for 30 minutes with the Ca^2+^ chelator BAPTA-AM before incubation with torin2+rapamycin. Pre-treatment with BAPTA-AM inhibited IL-6 secretion, indicating that Ca^2+^ is required for IL-6 secretion (torin2+rapamycin 1975±472% and bapta+torin2+rapamycin 484±137%) (Figure 6D). To assess if the torin2+rapamycin treatment could affect ER Ca^2+^, we performed Ca^2+^ imaging of drug-treated primary cultures loaded with the ratiometric dye Fura-2. To evaluate ER Ca^2+^ content, we treated primary cultures of astrocytes with the RyRs agonist 4-chloro-*m*-cresol (4-cmc) in Ca^2+^-free conditions, to avoid Ca^2+^ influx from the media [40]. Bath application of 4-cmc resulted in a rapid transient Ca^2+^ increase (peak 0.80±0.01 a.u., AUC 72±2 a.u.), followed by elevated intracellular Ca^2+^ levels (Figure 6D,E). The amplitude of this Ca^2+^ response is a measure of the Ca^2+^ stored in the ER. Stimulation with rapamycin increased the transient response (Figure 6D,E(I)) (peak 0.86±0.01 a.u.), in agreement with previous data reporting that rapamycin increases RyRs open probability without affecting the amount of Ca^2+^ stored in the ER (AUC 79±3 a.u.) [41]. Importantly, torin2 treatment caused a reduction of the peak induced by 4-cmc (Figure 6D,E(I)) (peak 0.765±0.008 a.u.), suggesting a depletion of Ca^2+^ in the ER. Stimulation of torin2+rapamycin treated astrocytes resulted in a significantly smaller peak delayed in time (peak 0.63±0.01 a.u.) (Figure 6D,E(I)). Moreover, both torin2 (AUC 53±4 a.u.) and torin2+rapamycin (AUC 36±2 a.u.) treatment also caused a decrease of the area under the curve suggesting a decrease in ER Ca^2+^ content or RyR expression (Figure 6D,E(II)).

### Combined Treatment with Torin2 and Rapamycin has a Small Temporary Effect on Mechanical Hypersensitivity

**Figure 7:**
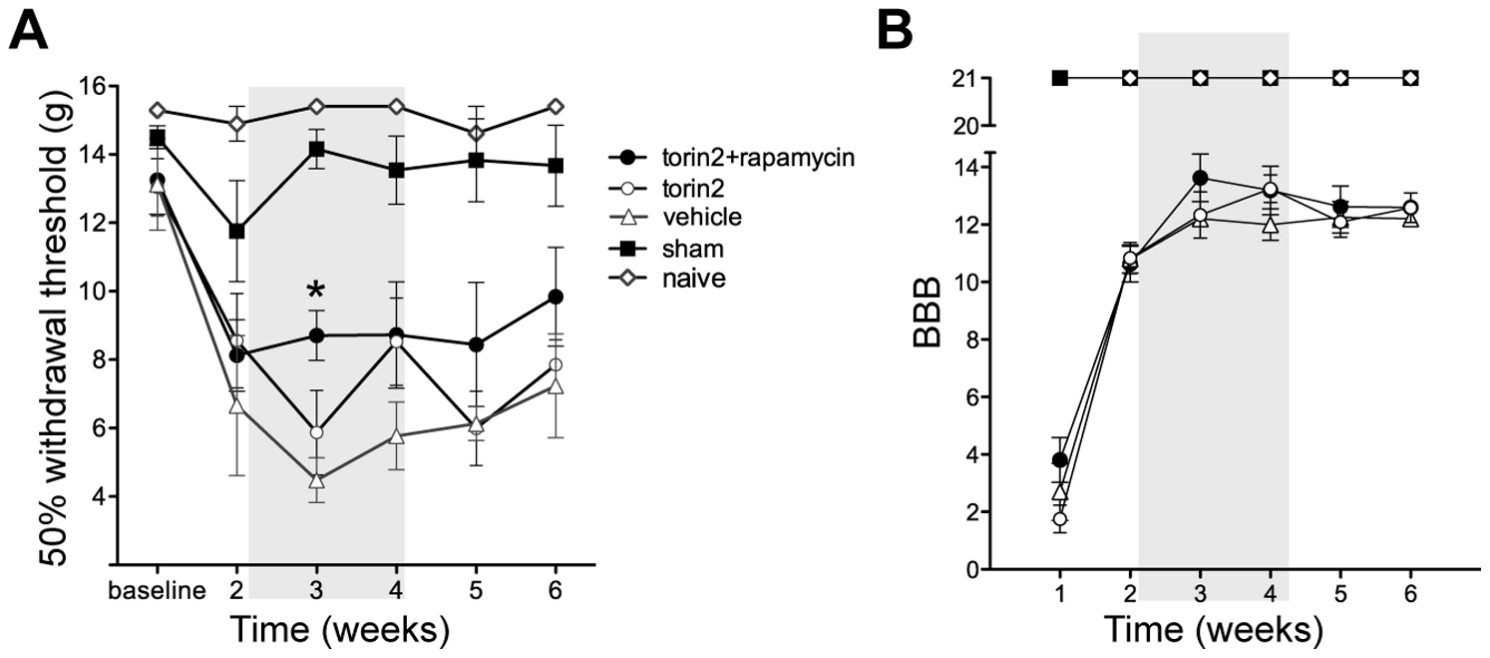
Combined treatment with torin2 and rapamycin improves mechanical hypersensitivity during treatment. Mechanical hypersensitivity and locomotion of injured animals assessed for 6 weeks after contusion using von Frey filaments and the BBB scale, respectively. The gray highlighted region represents the treatment period. **A**, Mechanical hypersensitivity expressed as changes in 50% withdrawal threshold (g) over time. Repeated measures two way-anova show a significant effect of torin2+rapamycin (p<0.05) in the treatment window. Using bonferroni post-test correction for multiple comparisons showed that torin2+rapamycin significantly reversed SCI-induced mechanical hypersensitivity at week three. (*p<0.05). **B**, Hindlimb locomotor function assessed by the BBB locomotor rating scale. (Naïve = 4, sham N = 6, pbs N = 6, torin2 N = 6, torin2+rapamycin N = 8).

In order to examine if torin2+rapamycin treatment has a positive effect on recovery from SCI, we treated injured animals daily from day 15 to day 29 after injury with torin2 alone or in combination with rapamycin. At this post-injury period the glia scar was already mature and the endogenous IL-6 expression of IL-6 was the same as sham animals. Even though both torin1 and torin2 strongly inhibit mTOR, we decided to use torin2 because it has a lower EC50, better bioavailability, and because we could develop a special formulation designed to enable oral administration of the drug [36]. Torin2 treatment alone did not have any effect on mechanical hypersensitivity or locomotion (Figure 7A,B). In contrast, dual treatment with torin2 and rapamycin had a small temporary effect on mechanical hypersensitivity during the treatment window (Figure 7A), thus indicating a beneficial role of the treatment.

## Discussion

Spinal cord astrocytes respond to injury by forming a scar that protects the healthy tissue and inhibits regeneration. Therefore, the induction of regeneration promoting factors, such as IL-6 in reactive astrocytes, may be beneficial for recovery after injury. We found that SCI induces IL-6 in reactive astrocytes and that IL-6 levels decrease concomitantly with maturation of the glial scar. We show that IL-6 production by astrocytes requires inhibition of the PI3K-mTOR pathway, together with increased cytosolic Ca^2+^ concentration. Regulation of these pathways is achieved by treating astrocytes with the PI3K-mTOR inhibitor torin2 together with the RyRs opening facilitator, rapamycin. To determine if torin2+rapamycin treatment is beneficial for recovery, we treated injured animals when the scar had already been formed. Treated animals showed a temporary improvement of mechanical hypersensitivity limited to the drug treatment period.

In order to determine the time course of IL-6 induction after SCI, we measured cytokine levels in plasma and spinal cords from injured rats. In agreement with previous reports [42], we detected increased IL-6 levels in both plasma and spinal cord parenchyma. Interestingly, even though plasma levels returned to baseline one week after injury, spinal cord IL-6 levels remained elevated. Using *in situ* hybridization, we found that astrocytes, neurons and microglia/macrophages are positive for IL-6 mRNA even though there is no circulating IL-6, indicating that these cells are the local producers of IL-6 in the phase following the first reaction to injury. Thus, given that IL-6 can be produced by different cells at different time points and the levels can be elevated either systemically or only locally, it is plausible that IL-6 plays different roles in pathology and repair depending on the time frame and localization of expression. Importantly, spinal cord expression of IL-6 returns to baseline once the scar is mature three weeks after injury, and thus any positive effect of IL-6 on axonal regeneration would no longer be in place. Hence, one strategy to promote recovery after SCI even after the formation of a glial scar would be to drive continued release of IL-6 to enhance IL-6-mediated positive effect on regeneration. However, this requires an understanding of the molecular mechanisms that regulate expression and release of IL-6 by astrocytes.

The PI3K-mTOR pathway is one regulatory element for expression of inflammatory cytokines in immune cells [43], and here we showed that it inhibits IL-6 expression in astrocytes. PI3K-mTOR signaling converges on AKT, and it has been demonstrated that active AKT has an inhibitory impact on inflammatory genes in immune cells [44]. The negative impact on cytokine production by AKT is mediated by inhibition of p38 and/or NF-κB activity [45], [46]. Astrocytes treated with the PI3K-mTOR inhibitor, torin2, have decreased AKT activity and increased MAPKs and NF-κB activities. Interestingly, it has been reported that p38 not only drives transcription of IL-6 by promoting NF-κB activity, but also stabilizes IL-6 mRNA [47]. Our data show that in astrocytes the IL-6 mRNA level is regulated by p38 and NF-κB activities with a molecular mechanism similar to what has been previously reported for immune cells. Even though PI3K-mTOR inhibition with torin2 increases IL-6 mRNA in astrocytes, we show that IL-6 protein is not secreted in drug-treated cultures. The lack of IL-6 in the supernatant may result from inhibition of mRNA translation or lack of activation of secretory pathways. It has recently been shown that soluble cytokines like IL-6 are synthesized in the ER, transferred to the Golgi, and then collected in small secretory vesicles that release their cargo upon stimulation by fusing with the plasma membrane [9]. Though not studied in astrocytes, Ca^2+^ buffering in mast cells completely blocks IL-6 secretion [48]. Interestingly, when we stimulated astrocytes with rapamycin or FK506, compounds, which normally increase open probability and leakage from RYRs on the ER, no increase in IL-6 secretion was found. However, when the treatment was combined with PI3K-mTOR inhibitors, IL-6 levels were readily detectable in the supernatant of astrocyte cultures. This finding demonstrates that IL-6 secretion in astrocytes requires both mRNA production and increased intracellular Ca^2+^. Additionally, comparing FK506 and rapamycin showed that FK506, in combination with torin2, is a stronger inducer of IL-6. This may be explained by the ability of FK506 to interact with calcineurin, releasing the inhibition of InsP_3_R in the ER, which can result in higher intracellular Ca^2+^ and therefore a stronger promotion of IL-6 secretion [16], [17].

When astrocytes are stimulated with the RyRs agonist 4-cmc, they release Ca^2+^ from the ER. We found that 4-cmc stimulation of rapamycin-treated astrocytes induced a greater response, compared to vehicle treated cells, thus confirming that rapamycin can potentiate the effects of 4-cmc by increasing the open probability of RyRs. Furthermore, 4-cmc stimulation of torin2 treated astrocytes induced a smaller response compared to vehicle. We hypothesize that torin2 treatment caused a depletion of Ca^2+^ stored in the ER, and therefore not only reduced the response to 4-cmc but also increased the cytosolic Ca^2+^ concentration. However, we suggest that the increased cytosolic Ca^2+^ concentration was not enough to induce RyRs opening via a Ca^2+^ -induced Ca^2+^ -release mechanism, because torin2-treated cells still responded to 4-cmc stimulation. Additionally, the torin2-induced increase in cytosolic Ca^2+^ concentration is not enough to induce IL-6 secretion. However, when rapamycin is added to torin2, the leakiness of RyRs is increased and the cells do not respond to 4-cmc stimulation. We suggest that the increase in cytosolic Ca^2+^ concentrations induced by torin2, in presence of rapamycin, is sufficient to cause the opening of the RyRs, thus depleting the ER from Ca^2+^ and inducing fusion of the IL-6 containing vesicles with the plasma membrane (hypothesis depicted in Figure S3). However, additional work is required in order to verify this hypothesis.

Our data show that IL-6 secretion from astrocytes requires PI3K-mTOR inhibition, and suggests that IL-6 mRNA is translated even when mTOR, the master controller of protein synthesis, is inhibited. mTOR has been shown to regulate CAP-dependent translation of transcripts with oligopyrimidine (TOP/TOP-like) motifs in the 5′-untranslated region (5′-UTR) [49]. The IL-6 5′-UTR does not match the characteristics of TOP/TOP-like mRNAs, when the classification criteria illustrated by Thoreen and co-workers are applied [50], suggesting CAP-independent translation. Interestingly, translation of stress-related proteins is also inhibited by the PI3K-mTOR pathway and is regulated through a non-well characterized CAP-independent mechanism [51].

Our data show that reactive astrocytes expressing IL-6 have a low mTOR activity, and three weeks after injury, when IL-6 level return to baseline, there is a concomitant increase in mTOR activity. An important implication of our findings is that by using torin2 and rapamycin, which block the PI3K-mTOR pathway and increases intracellular Ca^2+^ concentrations, it is possible to induce IL-6 release in reactive astrocytes. In light of the ability of IL-6 to promote axonal regeneration [52], [53], these drugs have the potential to contribute to recovery after SCI. We tested this hypothesis by treating rats subjected to SCI with torin2+rapamycin for two weeks starting two weeks after injury. The role of IL-6 in pain transmission is somewhat controversial and there are only a few reports on the role of IL-6 in pain induced by SCI [54]. We found a transient reversal in hypersensitivity during the period of drug administration. However, because the drugs were administered systemically we cannot exclude that the positive effect of treatment was mediated through regulation of other factors or cell types. Further studies are required in order to determine this in detail. However, the positive effect noted during the treatment window suggests that a prolonged or chronic treatment might be more beneficial after SCI. However, recent studies suggest that chronic treatment with mTOR inhibitors like rapamycin may lead to insulin resistance [55], [56]. However, a longer period of treatment may be enough to promote a more sustained reversal of mechanical hypersensitivity. Additional studies are necessary in order to determine the optimal treatment regiment.

In summary, we identified the PI3K-mTOR-Ca^2+^ pathway as the molecular mechanism that regulates IL-6 expression and secretion by astrocytes. We also identified that the combination of torin2 and rapamycin may have beneficial effects on sensory recovery after spinal cord injury, opening new possibilities for the development of a therapy.

## Supporting Information

Figure S1 **Control** ***in situ*** **hybridization with IL-6 sense mRNA probe.** **A**, Cartoon depicting: (I) the 7 mm long segments (rostral, caudal, epicenter) in which the injured spinal cord is dissected for analysis. (II) Regions of the spinal cord sections imaged. Dorsal horn (DH), dorsal column (DC), central canal (CC), white matter (WM) and ventral horn (VH). **B**, IL-6 *in situ* hybridization with sense IL-6 mRNA probe on sections from rostral, caudal, and epicenter segments from spinal cord of naive and injured animals 6 hours, 1, and 2 weeks post-injury. Scale bar = 200 μm. **C**, High magnification images from sections of the caudal segment shown in B from naive (20X) and 1 week injured spinal cord animals (20X and 40X). Scale bar = 20 μm for 20X and 40X. (N = 3 in each group)
(DOCX)

Figure S2 **IL-6 is not expressed by immune cells in white and gray matter. A**, Immunolabeling of IL-6 (green) and the microglia/macrophage marker OX-42 (magenta) of the white matter of the caudal segment from spinal cords 1 week after injury. **B**, Immunolabeling of IL-6 (green) and the microglia/macrophage ED1 (magenta) of the white matter of the caudal segment from spinal cords 1 week after injury. Scale bar = 20 μm. (N = 3 in each group)
(DOCX)

Figure S3 **Cartoon depicting the mechanism that regulates IL-6 secretion in astrocytes.** **A**, An astrocyte in naïve condition where PI3K-mTOR-AKT pathway is activated and inhibits IL-6 expression. **B**, Inhibition of the PI3K-mTOR-AKT pathway results in p38 activation and NF-κB-mediated transcription of IL-6. Torin2 treatment also decreases ER Ca^2+^ content suggesting an increase of Ca^2+^concentration in the cytosol. However, the increase is not sufficient to cause IL-6 secretion. **C**, A torin2 induced increase in cytosolic Ca^2+^ concentration is sufficient to cause opening of the RyR2 when rapamycin is also present in the cell. This causes a higher increase in Ca^2+^ concentration in the cytoplasm that is sufficient to induce IL-6 secretion.
(DOCX)
