# Supplementary material for: Interleukin-6 Secretion by Astrocytes Is Dynamically Regulated by PI3K-mTOR-Calcium Signaling
Source: PLoS One. 2014 Mar 25;9(3):e92649. doi: 10.1371/journal.pone.0092649 (PMC3965459; doi:10.1371/journal.pone.0092649)
Supplement: Figure S1 — Control in situ hybridization with IL-6 sense mRNA probe. A, Cartoon depicting: (I) the 7 mm long segments (rostral, caudal, epicenter) in which the injured spinal cord is dissected for analysis. (II) Regions of the spinal cord sections imaged. Dorsal horn (DH), dorsal column (DC), central canal (CC), white matter (WM) and ventral horn (VH). B, IL-6 in situ hybridization with sense IL-6 mRNA probe on sections from rostral, caudal, and epicenter segments from spinal cord of naive and injured animals 6 hours, 1, and 2 weeks post-injury. Scale bar = 200 μm. C, High magnification images from sections of the caudal segment shown in B from naive (20X) and 1 week injured spinal cord animals (20X and 40X). Scale bar = 20 μm for 20X and 40X. (N = 3 in each group) (DOCX) [file pone.0092649.s001.docx]

**Figure S1. Control *in situ* hybridization with IL-6 sense mRNA probe.**

**A**, Cartoon depicting: (I) the 7 mm long segments (rostral, caudal, epicenter) in which the injured spinal cord is dissected for analysis. (II) Regions of the spinal cord sections imaged. Dorsal horn (DH), dorsal column (DC), central canal (CC), white matter (WM) and ventral horn (VH). **B**, IL-6 *in situ* hybridization with sense IL-6 mRNA probe on sections from rostral, caudal, and epicenter segments from spinal cord of naive and injured animals 6 hours, 1, and 2 weeks post-injury. Scale bar=200μ. **C**, High magnification images from sections of the caudal segment shown in B from naive (20X) and 1 week injured spinal cord animals (20X and 40X). Scale bar = μ for 20X and 40X. (N=3 in each group)
