# Supplementary material for: Interleukin-6 Secretion by Astrocytes Is Dynamically Regulated by PI3K-mTOR-Calcium Signaling
Source: PLoS One. 2014 Mar 25;9(3):e92649. doi: 10.1371/journal.pone.0092649 (PMC3965459; doi:10.1371/journal.pone.0092649)
Supplement: Figure S2 — IL-6 is not expressed by immune cells in white and gray matter. A, Immunolabeling of IL-6 (green) and the microglia/macrophage marker OX-42 (magenta) of the white matter of the caudal segment from spinal cords 1 week after injury. B, Immunolabeling of IL-6 (green) and the microglia/macrophage ED1 (magenta) of the white matter of the caudal segment from spinal cords 1 week after injury. Scale bar = 20 μm. (N = 3 in each group) (DOCX) [file pone.0092649.s002.docx]

**Figure S2. IL-6 is not expressed by immune cells in white and gray matter.**

**A**, Immunolabeling of IL-6 (green) and the microglia/macrophage marker OX-42 (magenta) of the white matter of the caudal segment from spinal cords 1 week after injury. **B**, Immunolabeling of IL-6 (green) and the microglia/macrophage ED1 (magenta) of the white matter of the caudal segment from spinal cords 1 week after injury. Scale bar=μ. (N=3 in each group)
