# Supplementary material for: Interleukin-6 Secretion by Astrocytes Is Dynamically Regulated by PI3K-mTOR-Calcium Signaling
Source: PLoS One. 2014 Mar 25;9(3):e92649. doi: 10.1371/journal.pone.0092649 (PMC3965459; doi:10.1371/journal.pone.0092649)
Supplement: Figure S3 — Cartoon depicting the mechanism that regulates IL-6 secretion in astrocytes. A, An astrocyte in naïve condition where PI3K-mTOR-AKT pathway is activated and inhibits IL-6 expression. B, Inhibition of the PI3K-mTOR-AKT pathway results in p38 activation and NF-κB-mediated transcription of IL-6. Torin2 treatment also decreases ER Ca2+ content suggesting an increase of Ca2+concentration in the cytosol. However, the increase is not sufficient to cause IL-6 secretion. C, A torin2 induced increase in cytosolic Ca2+ concentration is sufficient to cause opening of the RyR2 when rapamycin is also present in the cell. This causes a higher increase in Ca2+ concentration in the cytoplasm that is sufficient to induce IL-6 secretion. (DOCX) [file pone.0092649.s003.docx]

**Figure S3. Cartoon depicting the mechanism that regulates IL-6 secretion in astrocytes.** **A**, An astrocyte in naïve condition where PI3K-mTOR-AKT pathway is activated and inhibits IL-6 expression. **B**, Inhibition of the PI3K-mTOR-AKT pathway results in p38 activation and NF-κB-mediated transcription of IL-6. Torin2 treatment also decreases ER Ca^2+^ content suggesting an increase of Ca^2+^concentration in the cytosol. However, the increase is not sufficient to cause IL-6 secretion. **C**, A torin2 induced increase in cytosolic Ca^2+^ concentration is sufficient to cause opening of the RyR2 when rapamycin is also present in the cell. This causes a higher increase in Ca^2+^ concentration in the cytoplasm that is sufficient to induce IL-6 secretion.
